# Victor William (1896-1978) and William Allen (1932-2010) Humpherson

**DOI:** 10.1038/s41415-024-8067-3

**Published:** 2025-02-14

**Authors:** Stanley Gelbier

**Affiliations:** Honorary Professor and Head of the Unit for the History of Dentistry, Faculty of Dentistry, Oral and Craniofacial Sciences, King´s College London, Guy´s Hospital, Tooley Street, London, London, UK

## Abstract

Father and son, Victor and William Humpherson, both became dentists, albeit in different eras. Although working in different branches of dentistry, both became important members of the profession. Victor was important in the early days of NHS general dental practice, finally as chairman of the Dental Estimates Board. William was area dental officer for Croydon, heavily involved in many developments in community dental services, especially in staff education and training. He was also an organist, playing a *Fantasia on three notes,* specially composed for the 1980 British Dental Association's centenary meeting. Both were nicknamed ‘Humph' but to avoid confusion, it is here only used to refer to William Allen.

## Key points

- Discusses the role of Victor William Humpherson in the evolution of NHS dentistry, eventually as chairman of the Dental Estimates Board.
- Discusses William Allen Humpherson's work in the community dental services and as an organist.
- Reminds readers that dentist-composer Wilfred Joseph specially wrote a piece for the organ to be played at the British Dental Association's centenary celebrations.

## Victor William Humpherson LDS

Victor was born in Bewdley, Worcestershire on 15 July 1896. He was the son of Harry Humpherson. World War I took him into the Worcester Regiment, later transferring to the Machine Gun Corps. He saw service in France, Egypt and Palestine.^1^ In 1916, he was wounded at the Somme but was one of the fortunate ones to survive.

Back in Civvy Street, he enrolled at the Royal Dental Hospital in London. In addition to his studies, Victor spent a lot of time playing sports, becoming captain of the cricket and hockey clubs. He represented both the United Hospitals and London University. Victor even played in 13 cricket matches for Worcestershire County.

In 1924, Victor gained his Licence in Dental Surgery (LDS). He stayed at the Royal as a house surgeon in the surgical department. He was then a demonstrator in conservation, teaching on Saturday mornings.

After his marriage to classmate Beryl Leal Allen LDS in 1926, they practised together at 25a Eccleston Street, London, SW1. They had two children: son William and daughter Patricia. Later, he also provided dental treatment at the Selfridges store on Oxford Street.

In the Second World War, Victor again become a soldier, volunteering for the Army Dental Corps (ADC) where he remained for the duration. His college friend, Wilfred Leigh Breese,^1^ stood in for him in Selfridges. Victor served in Sicily, Tunisia and Italy. He reached the rank of Lieutenant-Colonel, as Assistant Director of the ADC. After hostilities ended, he returned to his private practice: the NHS (National Health Service) had not yet arrived.

## Service for the British Dental Association

**Fig. 1:**
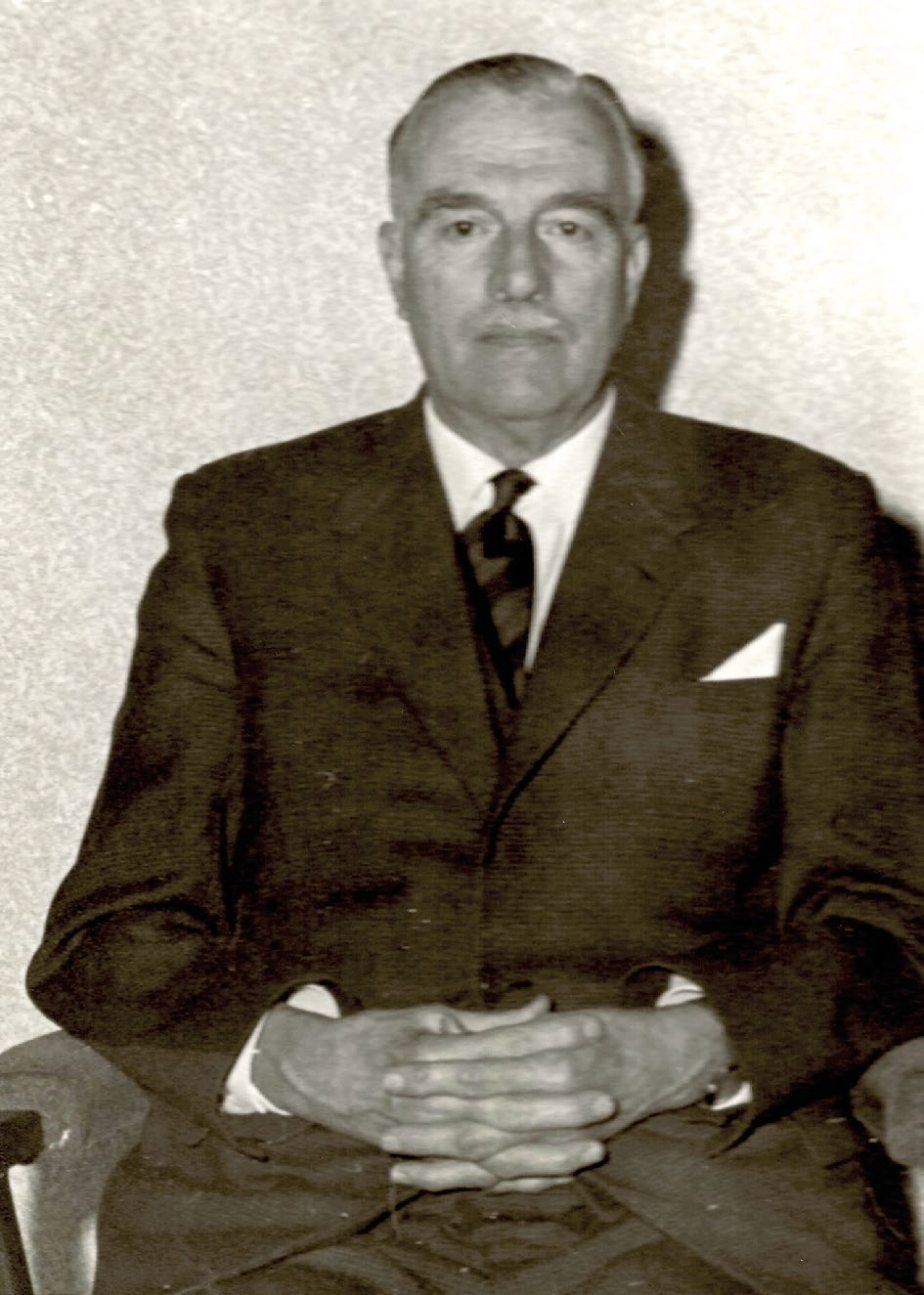
Victor Humpherson (image courtesy of BDA Museum)

Victor (Fig. 1) joined the British Dental Association (BDA) in 1926 and was soon involved with its activities. He organised demonstrations for the Association's Jubilee meeting in 1930 and that of the Empire Dental Meeting in 1936.

From 1935, he was secretary of the Metropolitan Branch, in which he remained for a number of years. This gave him membership of the Representative Board. In 1935, the Board elected him as vice chairman of the BDA Council - its highest body - which he remained for seven years.

Victor also had the inclination and found time for charitable activities. From 1945, he was treasurer of the Dentists Provident Society, remaining so for ten years. For five of those years, he was also a member of the Management Committee of the BDA Benevolent Fund.

## The coming of the NHS

After Victor left the army, he joined in the BDA's heated discussions on the proposed general dental services in the forthcoming NHS, but his views are unknown. From 1948, the general dental services formed an important part of the service. A Dental Estimates Board (DEB), forerunner of the Dental Practice Board, was established to control the payments to practitioners. As so often happens, there was much discussion as to whether it was good or bad for someone with knowledge of the BDA's workings and thought to go over to ‘the other side'. Victor did and was appointed as the DEB's vice-chairman. From 1955 to 1964, Victor was the chairman, working well with the BDA and the Department of Health, to which the DEB was accountable. In 1964, he was elected a life member of the BDA.

Victor died on 19 October 1978. He was survived by both children: Patricia Linley (1928-2008), district dietician for Tower Hamlets Health Authority in Whitechapel; and William (1932-2010), area dental officer for Croydon Area Health Authority.

## William Allen Humpherson BDS LDS DDPH LRAM FRCO

William (known as ‘Humph') was born in Eccleston Street, London on 24 March 1932. He was Victor's second child. Humph was first schooled at Font Hill Primary School, West Sussex. He then went to Tonbridge School in Kent. It was founded in 1553 by Sir Andrew Judde, a distinguished member of the Worshipful Company of Skinners, which assumed the governance of the School after his death. Interestingly, the Edwardian Chapel has a superb four-manual organ built by Marcussen of Denmark, regarded as one of the finest instruments in the country. One wonders if it influenced Humph's later choice of musical instrument to play.

After passing A-level exams, he entered the Royal Dental Hospital, 32 years after his father left. Humph gained his LDS in 1956 and Bachelor of Dental Surgery (BDS) in 1957. He joined the Dentists Register on 4 December 1956, where he was listed at the same address as his father: Tanglewood, Rowfant, Crawley, Sussex.

Humph did his two years National Service as a Surgeon-Lieutenant (Dental) in the Royal Navy, based in Portsmouth. He lived in a variety of places in Southern England, including Torquay, Salisbury and latterly at The White Cottage, Somerset Road in Reigate. He occupied several posts as a school dental officer. Humph worked for the Wiltshire School Dental Service in Salisbury under Don Middleton. To improve his knowledge and expertise, Humph joined the Bristol Diploma in Dental Public Health (DDPH) course on a part-time basis. His June 1972 course dissertation was on variations in susceptibility to caries with particular reference to twins and genes.^2^

**Fig. 2:**
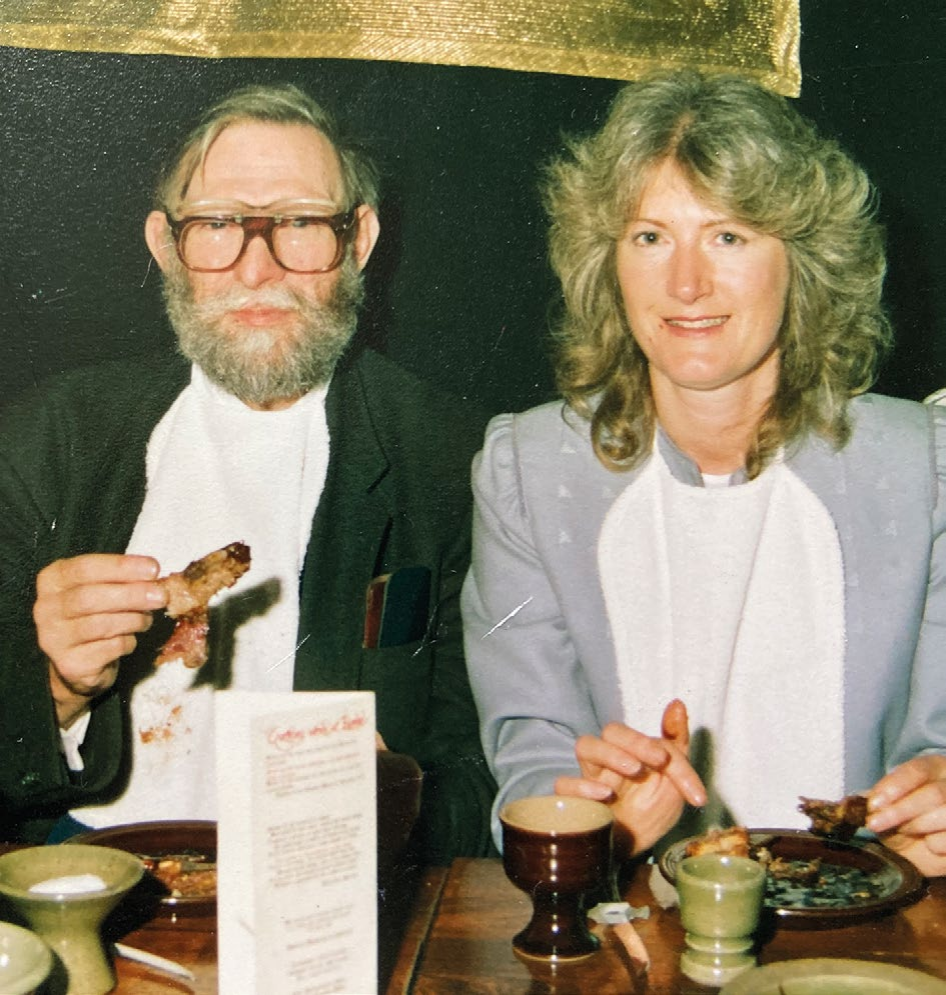
William Humpherson and Pam Usher (senior dental officer North-West Surrey) at a BDA community dental service group annual dinner held in Birmingham in 1991 (image courtesy of Pamela Usher)

In 1974, Humph became area dental officer of Croydon, managing the community dental services (CDS). For many years, he was based at its Lodge Road Dental Clinic, where he surrounded himself with hard-working and enthusiastic senior dental staff, including Ted Taylor, Alan Howe and Les Cheeseman. Humph enjoyed meetings of the Community Dental Services Group of the BDA (Fig. 2).

## Community dentistry

There are a number of references to Humph's community activities recorded in the BDA's John McLean Oral Archive.^3^ An interesting group discussion took place among community service dentists (Box 1) at BDA headquarters in October 2011. They examined developments which had taken place over the years in school and community services. An interesting comment came from Nigel Williams who joined Croydon's Service in 1986, who ‘[had] been sacked from the practice where [he] was an associate because [he] didn't cut 20 crowns a week'. He said the lovely family practice he had initially joined had been taken over by a ‘real go-getter', so he joined the Croydon CDS. Its district dental officer was ‘William Humpherson, an extraordinary gentleman, very, very, very brilliant in his own way. And very far sighted'. He said, long before officialdom caught up, that Humph was telling his staff to treat more physically and mentally disabled adults, as well as children. That was indeed ‘far sighted'. It was not easy, as the experience of many community dentists was only treating children. To convert to a totally different clientele required a different clinical skill set.

Humph encouraged his staff with internal support and training and sent some off for additional qualifications. Thus, when the change of the role of school to community services came in the 1970s, many seeds had already been sown.

Nigel said one of Humph's senior dental officers - Leslie Cheeseman^4^ - taught Nigel and other staff about the basics of relative analgesia. As he showed an interest, when Nigel joined the service full-time, Humph suggested he get further training from a course on relative analgesia arranged by Philip Bristow in Portsmouth. Nigel was astounded by what he learned, leading to a major, longstanding interest. It was an example of Humph sending the right person to the right course to better themselves and the service they provided.

When Nigel joined the CDS, Humph had two senior dental officers: Les Cheeseman and Ted Taylor. They had both been general dental practitioners and both were endowed with much common sense. Their wealth of experience was very willingly shared with the staff, much encouraged by Humph.

Sometimes, it was just to gain further knowledge and experience, but often, Humph sent staff for formal education and training. For example, he encouraged and ensured payment for Nigel to join a Master of Science course at King's. For personal reasons, the latter only completed part of that course, but says he learned about many aspects of dental and sociological life ‘which have always stood me in good stead'. He later joined the DDPH course at the Eastman and gained even more. That was typical Humph, assessing the needs of staff and sending them on an appropriate course.

For a period, Humph was a part-time lecturer in community dentistry at King's College Dental School, supporting postgraduate student projects.

In many ways, to some people who didn't know him, Humph seemed slightly eccentric. However, he had a heart of gold and a passion for the service he managed, his staff and their patients.

Box 1 Participants in a John McLean Archive discussion on 4 October 2011

- Leslie Cheeseman
- Stanley Gelbier
- Sandra Halford
- Christine Holmberg
- Alan Howe
- Roy Jackson
- Mitzi Macey-Dare
- Robin Rippon
- Astrid Stockel
- Pamela Usher
- Jacob Van den Berg
- Jerry Walsh
- Nigel Williams

## Humph and planning

Humph was very keen to keep himself up to date and to ensure services were appropriate. He and his colleagues in the South West Thames health region carried out an oral health survey of 500 children aged five, 490 aged 12 and 480 15-year-olds. They wanted to compare their findings with the 1973 national survey.^5^ The dental officers thought that although the latter provided much valuable data, it was insufficient for planning their local services. They therefore delegated ten community dental officers to carry out the regional survey, first sending them to Guy's Hospital to be trained and calibrated by Professor Frank Ashley.

In 1973, they found children in the region had better oral health than those in London and the South East.^6^ In total, 65% of their five-year-olds had never experienced decay compared with 33% of the latter group. Additionally, 33% needed treatment (versus 57%). By age 12, the DMFT (decayed, missing and filled teeth) score was 6.4 (versus 8.1). There was some gingival inflammation in 27% (versus 54%). Humph was able to put this information to use for planning, appropriately directing staff and other resources to where needed.

## Passion for the organ

Outside of dentistry, Humph was passionate about music, especially the organ, which he played. Remarkably, Humph only started learning music at the age of 15, not having come from a musical family. He quickly attained a very high level of musical capability. He was a licentiate of the Royal Academy of Music (LRAM) and fellow of the Royal College of Organists (FRCO).

Humph was often invited to speak to community vocational trainees as an example of someone who did well in the profession but still found time for outside interests. He talked about the music of Bach and treated them to recordings of him playing Bach's music on the very organs in Europe on which Bach had played it.

**Fig. 3:**
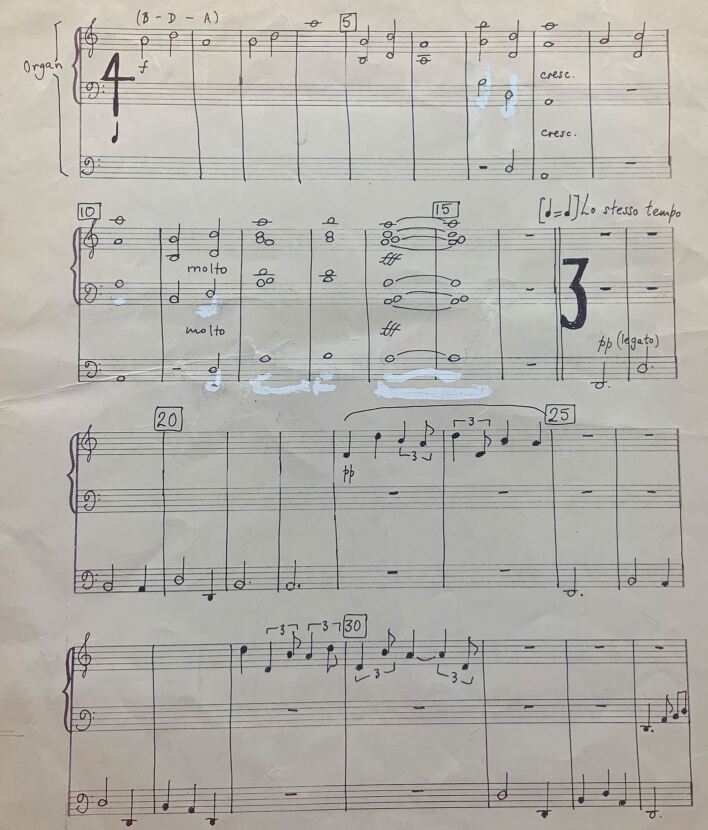
Beginning of *Fantasia on three notes* (image courtesy of Ed Humpherson, from his father's archive)

On 10 July 1980, he played a Fantasia on three notes for the BDA's Centenary meeting at the Royal Festival Hall.^7^ It had been specially composed for the occasion by famed dentist-musician Dr Wilfred Josephs BDS DMus.^8^ Based on the three musical notes of ‘B, D and A' it represented the embodiment in sound of the Association's monogram (Fig. 3).

On 19 January 1957, he married Rosemary Elizabeth FitzGerald Arbuthnot (daughter of Hugh FitzGerald Arbuthnot and Kathleen Phyllis Sheppard) in Ashbury, Oxfordshire. They had met at Pigott's Music Camp and shared a lifelong love of music. They had four children: Robert William (born 1958), Susan Linley (1960; later Hodgson), Michael Hugh (1962) and Edward Allen (1970). Only Susan followed him into dentistry/community dentistry, having qualified at Bristol Dental School.

William died peacefully in Reigate, Surrey after a short illness on 24 August 2010, leaving behind Rosemary, their four children and seven grandchildren. He was buried at All Saints Churchyard in Crawley, Sussex. A notice of his death in The Times described him as a campaigner for patients' interest and a mind of endless curiosity.
